# Plasma neurofilament light chain levels modulate the effects of cognitive intervention: Evidence from a PICMOR randomized controlled trial

**DOI:** 10.1002/alz70861_108730

**Published:** 2025-12-23

**Authors:** Mihoko Otake‐Matsuura, Hikaru Sugimoto, Takuya Sekiguchi, Masato S. Abe, Kumi Watanabe Miura, Seiki Tokunaga, Shoshin Akamine, Taishiro Kishimoto, Takashi Kudo

**Affiliations:** ^1^ RIKEN, Tokyo Japan; ^2^ Japan Society for the Promotion of Science, Tokyo Japan; ^3^ Doshisha University, Kyoto Japan; ^4^ Osaka University, Toyonaka Japan; ^5^ Keio University, Tokyo Japan; ^6^ Iseikai International General Hospital, Osaka Japan

## Abstract

**Background:**

Although individual differences in neuronal states are evident, it is only in recent years that these factors have been incorporated into intervention research. Progress in analytical technologies applied to hematological examinations have made it increasingly feasible to assess neuronal states in a more accessible and efficient manner. This study utilized advanced analytical approaches to determine whether cognitive intervention outcomes are influenced by the concentration of blood‐derived biomarkers, such as neurofilament light chain (NfL), since NfL in plasma is considered a promising biomarker for neurodegenerative conditions, notably including Alzheimer's disease.

**Method:**

The intervention protocol implemented in this study was Photo‐Integrated Conversation Moderated by Robots (PICMOR), a group‐based conversational approach shown to promote verbal fluency in older populations. In order to assess the potential modulation of intervention effects by individual neuronal states, we performed a randomized controlled trial (UMIN Clinical Trials Registry number: UMIN000036599) to examine how longitudinal changes in cognitive performance, including verbal fluency, correlate with baseline NfL levels. Plasma levels of NfL were assessed twice on the Simoa platform, employing Simoa NF‐Light Advantage kits and the Simoa HD‐1 Analyzer instrument (Quanterix Corporation).

**Result:**

Positive effects of the PICMOR intervention on verbal fluency were observed in individuals with lower NfL levels, suggesting a relatively preserved neuronal state. The intervention effect was positively significant at the 5% level for individuals with NfL less than 12(pg/mL). In contrast, individuals with higher baseline NfL levels experienced negative effects on verbal fluency from the intervention. The intervention effect was negatively significant at the 5 % level for individuals with NfL more than 28(pg/mL).

**Conclusion:**

Our results indicate that the effects of cognitive intervention are modulated by the plasma NfL concentration. Consequently, it is recommended that future intervention studies incorporate the neuronal condition of participants to assess the effects of the intervention.

